# Genotype–Phenotype Correlations in Relation to Newly Emerging Monogenic Forms of Autism Spectrum Disorder and Associated Neurodevelopmental Disorders: The Importance of Phenotype Reevaluation after Pangenomic Results

**DOI:** 10.3390/jcm10215060

**Published:** 2021-10-29

**Authors:** Carla Lintas, Roberto Sacco, Alessia Azzarà, Ilaria Cassano, Fiorella Gurrieri

**Affiliations:** Laboratory of Medical Genetics, University Campus Bio-Medico of Rome, 00128 Rome, Italy; r.sacco@unicampus.it (R.S.); a.azzara@unicampus.it (A.A.); i.cassano@unicampus.it (I.C.)

**Keywords:** autism spectrum disorder, neurodevelopmental disorders, exome sequencing, phenotype reevaluation, ASD, NDDs

## Abstract

ASD genetic diagnosis has dramatically improved due to NGS technologies, and many new causative genes have been discovered. Consequently, new ASD phenotypes have emerged. An extensive exome sequencing study carried out by the Autism Sequencing Consortium (ASC) was published in February 2020. The study identified 102 genes which are de novo mutated in subjects affected by autism spectrum disorder (ASD) or similar neurodevelopmental disorders (NDDs). The majority of these genes was already known to be implicated in ASD or NDDs, whereas approximately 30 genes were considered “novel” as either they were not previously associated with ASD/NDDs or very little information about them was present in the literature. The aim of this work is to review the current literature since the publication of the ASC paper to see if new data mainly concerning genotype–phenotype correlations of the novel genes have been added to the existing one. We found new important clinical and molecular data for 6 of the 30 novel genes. Though the broad and overlapping neurodevelopmental phenotypes observed in most monogenic forms of NDDs make it difficult for the clinical geneticist to address gene-specific tests, knowledge of these new data can at least help to prioritize and interpret results of pangenomic tests to some extent. Indeed, for some of the new emerging genes analyzed in the present work, specific clinical features emerged that may help the clinical geneticist to make the final diagnosis by associating the genetic test results with the phenotype. The importance of this relatively new approach known as “reverse phenotyping” will be discussed.

## 1. Introduction

With the recent development of NGS technologies, ASD genetic diagnosis has dramatically improved, and many new genes associated with ASD have been discovered. Consequently, new ASD phenotypes have emerged. Therefore, it seems that we are entering a new era, in which the ASD diagnostic path definitely benefits from a genetic work-up, as it can lead to a precise definition of etiology, family counselling, prognosis forecasting, and gene-based stratification, hoping that targeted therapies are eventually developed. We need to be aware that the clinical presentation of ASD (also in terms of physical traits) in some instances drives crucially the genetic testing, which is often a multi-step process. Bearing this in mind, and by reviewing the emergent ASD genes and phenotypes, we will outline some practical guidelines that can be useful for geneticists and neuropsychiatrists to implement the genetic diagnosis of patients with ASD.

In February 2020, the most extensive exome sequencing study on autism spectrum disorders (ASD) and related neurodevelopmental disorders (NDD) was published by Satterstrom and colleagues [1]. A total of ~35,500 samples were sequenced including ~21,000 family-based samples and 14,365 case-control samples. The authors analyzed de novo variants and observed a highly significant 3.5-fold enrichment of de novo protein truncating variants (PTVs), which are considered the most pathogenic variants, compared to a non-significant 1.2-fold enrichment of inherited protein truncating variants. Furthermore, de novo missense variants belonging to the most pathogenic category also displayed a 2.1-fold significant enrichment. The authors identified 102 genes, of which 53 were associated with ASD only, whereas the remaining 49 were related to ASD and other neurodevelopmental disorders (NDDs). The 102 genes were classified into four functional categories: gene expression regulation (58 genes), neuronal communication (24 genes), cytoskeleton (9 genes), and other (11 genes). Among the 102 genes, 60 had not been discovered by previous exome sequencing studies and, within them, 30 were considered by the authors “truly novel” (Table 1), as little or no information for these genes was present in the literature at the time of publication release. Indeed, none of these novel genes were associated with an OMIM phenotype at that time (https://www.omim.org/ (accessed on 1 July 2021)), but six of them were listed in OMIM afterwards. The aim of this paper is to review the current literature for new information and data concerning these “truly novel” defined genes and their associated phenotype. Indeed, our ultimate goal is to delineate genotype–phenotype correlations for these newly emerging genes in order to help the clinical geneticist to address the (a) appropriate genetic test if possible and above all (b) to interpret pangenomic results in light of the phenotype in patients with non-syndromic ASD.

## 2. Materials and Methods

We introduced the following key words in Pubmed for each of the 30 truly “novel genes”: (i) name of the gene; (ii) name of the gene AND autism; (iii) name of the gene AND neurodevelopment; (iv) name of the gene AND intellectual disability. We selected all papers not mentioned by Satterstrom and colleagues [1] as they were published afterwards. 

## 3. Results

We found new information concerning the correlation between the “novel genes” and emerging phenotypes for 6 out of the 30 novel genes. The list of the 30 novel genes and new data concerning emerging genotype–phenotype correlations are summarized in Table 1 and Table 2, respectively. Novel genes for which new data were available after the publication of Satterstrom and colleagues [1] are highlighted in bold. For the remaining 24 “novel genes”, no recent literature data were available.

### 3.1. The RORB Gene

The *RORB* gene (OMIM*601972) located on chromosome 9q21.13 is a transcription factor expressed in immature neurons where it regulates neural differentiation and migration. Two isoforms are known, RORß1 and RORß2, which are differentially expressed: the first one predominately in the retina and pineal gland, whereas the other one in more central nervous system (CNS) regions including the cortex, spinal cord, and pituitary [13]. Copy number variants (CNVs) involving deletions of the *RORB* gene have been described to be associated with epilepsy and mild intellectual disability by Baglietto and colleagues [14]; intellectual disability, speech delay, epilepsy, and dysmorphic traits by Boundry-Labis and colleagues [15] and developmental delay and ADHD by Tug et al. (2018). The most common facial dysmorphisms reported in these studies were hypertelorism, smooth philtrum, and thin upper lip [16]. The smallest overlapping deleted region (750 kb long) included four genes of which *RORB* was considered the strongest candidate. The causal role of *RORB* was recently confirmed by the identification of missense, nonsense SNVs (single nucleotide variant) [17] and intragenic deletions involving *RORB* in patients with epilepsy (idiopathic generalized susceptibility to 15 MIM#618357), sometimes in comorbidity with intellectual disability. A recent report [2] describes a neurodevelopmental clinical phenotype associated with a de novo *RORB* SNV in a patient affected by intellectual disability, attention deficit hyperactivity disorder (ADHD), and eyelid myoclonia with absences (Table 2). The latter is considered a form of generalized epilepsy. No previous reports have associated SNVs in the *RORB* gene with ADHD. The authors conclude their report stating that the functional role of RORB perfectly fits with the observed phenotype: generalized epilepsy as well as ADHD, which both involve a wide dysfunction of cortical and subcortical areas rather than a focal brain impairment. In our NDD patient cohort, we described a girl affected by a severe non-verbal form of ASD with a small de novo CNV (38 Kb in size) encompassing an intragenic duplication of the *RORB* gene [4]. At the time of the diagnosis, she was only three years old and was not affected by epilepsy, but she had important hand stereotypies and dysmorphic features. Interestingly, the girl was the third child of an ASD multiplex family and shared some inherited CNVs with the two eldest brothers but not the one encompassing *RORB* which arose de novo. Indeed, the two brothers were affected by mild NDDs: the first one had an expressive language impairment, whereas the second one displays an unusual form of ASD, characterized by very intense visual sensory self-stimulation and stereotypic behaviors. A recent report [3] identified novel inherited heterozygous *RORB* variants in fourteen individuals belonging to four families: eleven of them were affected by epilepsy, one had intellectual disability, and two were unaffected. Epilepsy was focal or generalized in five patients, whereas six patients had both types. Cognitive difficulties ranging from individuals having learning difficulties at school to mild or severe intellectual disability were reported in seven out of eleven patients. In summary, it appears that *RORB* variants are mainly associated with different forms of epilepsy (including eyelid myoclonia with absence epilepsy, generalized epilepsy, and occipital epilepsy), often in comorbidity with neurodevelopmental disorders such as intellectual disability, ADHD, and ASD. However, sporadic cases of *RORB* variants have also been described in association with severe forms of ID only [3] and ASD [4].

### 3.2. The PRR12 Gene

The *PRR12* gene (OMIM*616633) encodes a proline-rich protein nuclear factor involved in neural development, and it has a possible DNA binding activity and a chromatin remodeling role. Cordova-Fletes and colleagues [18] reported on a de novo balanced translocation disrupting the *PRR12* gene in a girl affected by intellectual disability and neuropsychiatric alterations. More recently, de novo loss of function SNVs have been reported in three patients affected by neurodevelopmental disorders and iris abnormalities [19]. All patients had global developmental delay, intellectual disability, dysmorphic traits, and eye anomalies, whereas variable clinical features includes skeletal anomalies, hypotonia, autism, and anxiety. Common eye anomalies were stellate iris pattern and iris coloboma. These findings were recently confirmed by Chowdhury and colleagues [5] who described other 21 individuals with PRR12 variants including twelve frameshift, six nonsense, one splice site, two missenses, and one with a gross deletion. All patients were affected by neurodevelopmental disorders including intellectual disability (91%), speech delay (88%), and motor delay (83%). Eye defects were also common and included globe defect (17%), coloboma (29%), and visual impairment (77%) and also structural defects as anophtalmia, microphtalmia, and optic nerve and iris abnormalities. Neuropsychiatric features included autism, ADHD, aggression, anxiety, stereotypes, and repetitive behavior. Moreover, other additional common defects included hypotonia (61%), heart defects (52%), growth failure (54%), and kidney anomalies (35%). However, recent findings by Reis and colleagues [6] describe individuals encompassing dominant variants in *PRR12* affected by unilateral or bilateral complex microphtalmia without a neurodevelopmental phenotype. The cohort described by Reis and colleagues consisted of five individuals with asymmetric ocular phenotypes [6]. Only three of them had variable degree delay, but two had normal development and cognition, and none were affected by neuropsychiatric disorders. Additional features included short stature and dysmorphic faces, which were present in some individuals. In summary, *PRR12* SNVs are associated to a new multisystemic disease characterized mainly by eye abnormalities and almost always by a neurodevelopmental phenotype.

### 3.3. The SATB1 Gene

The *SATB1* gene (OMIM *602075) encodes for a transcription factor involved in development and in T cell maturation. Recently *SATB1* disrupting de novo pathogenetic variants has been identified in patients affected by NDD, suggesting a role for this gene also in neurodevelopment [1,20]. Den Hoed and colleagues [7] have just published a case series of 42 individuals with pathogenetic or likely pathogenetic *SATB1* variants. Of these variants, twenty-eight were de novo, three were inherited from an affected parent, two were inherited from unaffected parent (reduced penetrance), five were probably due to parental mosaicism, and for the last four, inheritance could not be established. Thirty individuals carried missense variants that clustered in the two CUT1 and CUT2 DNA-binding domains. On the other hand, PTVs were present in 10 subjects (two nonsense, seven frameshift, and one splice) and were localized throughout the entire gene. The remaining two patients had two small gene deletion variants. Interestingly, the authors observed a clear genotype–phenotype correlation: individuals carrying missense variants were more severely affected than individuals carrying protein-truncating variants (PTVs). Indeed, 57% of patients carrying a missense variant were affected by severe intellectual disability, which was not observed for any individuals carrying PTVs. Other common clinical features were (i) visual abnormalities such as myopia, amblyopia, divergent squint, and strabismus, and (ii) dental anomalies such as small, malpositioned, or widely spaced teeth. In addition, other severe clinical features as spasticity, hypotonia, and epilepsy were significantly more common in patients with missense variants than in patients with PTVs. These observations were confirmed by the authors using a partitioning around medoids clustering algorithm [21] based on 100 features derived from standardized clinical data: the individuals were clustered into two separate clinical groups, one with PTVs and the other with missense variants [7]. Computational analysis of facial features also supported the existence of two separate clinical groups: patients with missense variants had a different facial gestalt from individuals carrying PTVs. Overlapping facial features included facial asymmetry, prominent ears, puffy eyelids, and low nasal bridge. Furthermore, the authors characterized functionally some of these missense and PTV variants using HEK293T/17 cells. Results from in vitro assays confirmed that the two classes of identified variants (missense and PTVs) are responsible of distinct pathophysiological mechanisms associated to *SATB1* NDDs. Indeed, functional assays (based on cellular localization, transcription activity, and overall chromatin binding and dimerization capacity) using cells transfected with some of the identified missense variants localized in the CUT1 and CUT2 DNA binding domains clearly demonstrated a stronger binding of the proteins encoded by *SATB1* missense variants to their downstream targets compared to the wild type protein. Some of the identified PTVs localized in the last exon of *SATB1* were predicted to escape non-sense mediated mRNA decay (NMD), a quality control mechanism that selectively degrades mRNAs harboring premature termination. The prediction was also confirmed by functional assays, and the selected PTVs were further assessed with the same functional assays used for missense variants, showing remarkable differences between the two distinct variant classes with respect to protein localization and their ability to repress transcription. Protein stability and protein SUMOylation were assessed only in some of the selected PTVs. In summary, this work demonstrated the existence of two different classes of *SATB1* pathogenetic variants (missense and PTVs) associated with two clinically distinct NDDs and distinct pathophysiological mechanisms (haploinsufficiency for PTVs and deletions and altered transcriptional activity for missense variants). Furthermore, this work highlights the importance of combining clinical data, functional assays, in silico models, and genetic data in order to characterize and fully understand the pathophysiological mechanisms underpinning *SATB1*-associated NDDs. 

*SATB2* variants have been previously associated with SAS, *SATB2*-associated syndrome (OMIM#612313), a similar neurodevelopmental disorder. Indeed, Satb1 and Satb2 are both transcription factors involved in the processes of cortex development and maturation of neurons. However, unlike for *SATB1*, no differences could be discerned in the range or severity of phenotypes between individuals with clear loss-of-function mutations and those with missense variants, supporting haploinsufficiency as the common pathogenic mechanism for *SATB2* [22]. As expected, *SATB1* and *SATB2* patients display very strong overlapping phenotypes. However, subtle differences in the neurological phenotype have been reported: absence of speech and drooling seem to be more common in patients with SATB2 variants, whereas epilepsy seems to be more common in patients with *SATB1* variants, especially in patients with missense variants, as previously discussed [7].

### 3.4. RFX Family Genes

The *RFX3* gene (OMIM*601337) is listed among the 31 novel genes related to ASD and associated NDDs. This gene belongs to a family of transcription factors highly expressed in the developing and adult brain. Other members include *RFX1*, *RFX4* (OMIM*603958), and *RFX7* (OMIM*612660) genes. De novo and inherited deleted CNVs encompassing *RFX3* have been identified in a girl and in seven other patients all affected by ID, ASD, and behavioral problems [23]. A deletion of *RFX3* has also been reported in a patient affected by schizophrenia [24]. On the other hand, no neurodevelopmental phenotype has been described associated with *RFX4* and *RFX7* variants. Indeed, a very recent report by Harris and colleagues [8] describes a cohort of 38 individuals affected by similar neurodevelopmental syndromes with mostly de novo variants (30/33 variants) in *RFX4* and *RFX7* genes in addition to the already known *RFX3* causal gene. Variants in all three genes included missense variants (thirteen), frameshift variants (eight), nonsense variants (five), splicing variants (two), and deletions (five) (Table 1). For all three genes, most missense variants were located in the DNA-binding domain (DBD). In addition to the DBD, RFX3 and RFX4 proteins also have three dimerization domains (DD) in which several missense, frameshift, and deletion variants of *RFX3* and *RFX4* fall. *RFX* transcription factors bind to a X-box consensus motif, which was found by the authors in some neuronal specific enhancers and/or promoters of autism risk genes (such as *AP2S1*, *KDM6B*, *ANK2*, *NONO*, and *MYT1L*). All identified variants except for one were absent from the gnomAD human database (https://gnomad.broadinstitute.org (accessed on 1 July 2021)). In addition, the majority of variants (20 out 33) identified were predicted to cause protein truncation or gene deletion, supporting a loss of function or haploinsufficiency model. In relation to the remaining missense variants, eleven out of the 13 were predicted to be deleterious by at least four of six algorithms and two by two algorithms. Functional analysis of five non truncating variants using HeLa cells resulted in a consistent decrease in *RFX3* expression level affecting protein stability for the majority of them. Individuals affected by pathogenic or likely pathogenic variants in one of the three *RFX* transcription factor genes identified in this study shared a common and peculiar behavioral phenotype characterized by sensory auditory hypersensitivity and impulsivity. This unique behavioral phenotype was present in nearly all individuals. In addition to such distinct behavioral phenotype, *RFX*-affected individuals have ID/global developmental delay with ASD and/or ADHD. In general, individuals with *RFX4* or *RFX7* are more severely affected than individuals with *RFX3* variants. Furthermore, *RFX7* affected individuals all have language delay and ID and are less commonly affected by ASD or ADHD. On the other hand, dysmorphic facial features, seizures, and neuroimaging findings, though widely reported in affected patients, do not seem to be specific for any of the three *RFX* genes. The mechanism by which RFX variants affect neurodevelopment and cause a neurodevelopmental phenotype is not clear yet. A study by Sun and colleagues [25] has shown an enrichment of RFX motifs in acetylated signals in ASD brains compared to controls. Hence, additional studies should aim to identify the gene targets of RFX transcription factors and to clarify their pathophysiological mechanism.

### 3.5. The GRIA2 Gene

The *GRIA2* gene (OMIM*138247) encodes for one of the four subunits (GluA1-A4) of the AMPA glutamate receptor, a ligand-gated ion channel playing an important role in excitatory synaptic transmission in the central nervous system. Specifically, the GluA2 subunit encoded by *GRIA2* regulates calcium permeation and voltage rectification. Few reports have described an association between *GRIA2* variants and neurodevelopmental disorders. Currently, it is listed in OMIM as responsible for the NEDLIB phenotype (neurodevelopmental delay language impairment and behavioral disorders). Hackmann and colleagues [26] described a patient affected by ID, severe speech delay, gait abnormalities, and abnormal behavior (hyperactivity, attention deficit, and aggressive behavior) with a small de novo deleted CNV encompassing the *GRIA2* gene. A very recent report by Alkelai and colleagues [9] describes a young girl affected by childhood onset schizophrenia bearing a de novo stop *GRIA2* variant (p.Glu508Ter). Additional clinical features included autism spectrum disorder, epilepsy, and obsessive–compulsive disorder. The most extensive work on *GRIA2* genotype–phenotype correlations has been recently carried out by Salpietro and colleagues [10], who describe a cohort of 28 unrelated patients with de novo heterozygous *GRIA2* variants. In total, they found 24 de novo *GRIA2* SNVs (including eighteen missense, two splice site, one in-frame deletion, one stop-gain, and two frameshift) and three de novo 4q32.1 microdeletions. The authors performed functional assays in order to characterize several missense variants found in their cohort. When they expressed missense variants in HEK293T cells, they found a decrease in agonist current mediated by these “mutant subunits” compared to wild-type channels. Furthermore, co-expression of mutant or wild-type GluA2 subunits together with wild-type GluA1 showed significant differences in KA (kainic acid)-evoked current amplitude. Specifically, five missense variants significantly decreased the KA-evoked current amplitude compared to the wild-type variant, and some also increased calcium permeability. The functional characterization of *GRIA2* variants explain the neurological phenotypes observed in most patients very well. These include Rett-Syndrome-like features (regression, stereotyped hand movements, and screaming episodes), gait abnormalities including ataxia and dyspraxia, abnormal sleep rhythm, and irregular breathing patterns with hyperventilation episodes. Progressive microcephaly was also observed (in 14% of patients), though it was not as common as in Rett syndrome patients. In addition, seizures or developmental epileptic encephalopathy (DEE) were also reported in about 40% of patients, usually arising very early in life (within the first six months of life). These same neurological features are also reported in patients with variants in other genes encoding for AMPAR/NMDAR subunits as *GRIA1*, *GRIA3*, and *GRIA4*. With respect to the main core phenotype, all patients had language impairment, which was severe in most cases. Other frequently reported neurodevelopmental disorders included ID and ASD. Some patients with GRIA2 variants also display progressive brain atrophy (mainly cerebellar) and white matter anomalies [10]. In summary, patients with GRIA2 pathogenetic variants display a broad spectrum of neurodevelopmental disorders, which makes it difficult for the clinical geneticist to address a specific test. However, the striking neurological features that characterize these patients subtly imply that the causative gene must be involved in synaptic transmission and/or brain plasticity, giving a clue to the clinical geneticist on where to search, at least in the first place. In support of this concept, a recent work by Peng and colleagues [27] aimed at finding shared genetic pathways between epilepsy and autism by means of a biostatistical approach: they combined genetic and phenotypic information and found a list of candidate causal genes shared by the two brain disorders that could be screened in the first place in patients where they co-occur. 

### 3.6. The TAOK1 Gene

The *TAOK1* gene (OMIM*610266) encodes for the serine/threonine protein kinase TAO1, which is highly expressed in the brain, where it regulates many cellular processes. The evidence for this gene in NDDs was very limited before the release of Satterstrom’s paper in May 2020 [1], with only a report by Xie and colleagues [28] about a patient with a de novo microdeletion at 17q11.2 associated with developmental delay, short stature, microcephaly, and dysmorphic features. However, two papers describing two cohorts of patients with *TAOK1* variants and their associated phenotypes have been published recently [11,12]. Dulovic-Mahlow and colleagues [11] reported eight individuals with *TAOK1* variants: four missense variants (p.Glu17Gly, p.Lys298Glu, p.Asp305Ala, p.Ser111Phe), three nonsense variants (p.Glu781*, p.Gln544*, p.Glu830*), and one frameshift (p.Leu790Phefs*3) variant. All eight individuals were affected by developmental delay, six by muscular hypotonia, and four by intellectual disability, and five had dysmorphic facial traits. The authors further functionally characterized the frameshift variant p.Leu790Phefs*3. For this variant, they showed decreased mRNA levels compared to controls in blood cells as well as in fibroblast-derived cells from the same patient. Additionally, the mutant fibroblasts were unable to express phosphorylated TAO1 kinase and tau protein and displayed altered mitochondrial morphology. Knock-down experiments of the ortholog gene *Tao 1* in *Drosophila* confirmed a role for this gene in neurodevelopment: mutant flies exhibited altered morphology of the ventral nerve cord and of neuromuscular junctions. Additionally, a decrease in the size of motor neurons’ axons and altered mitochondria distribution were reported in the mutant flies. The recent work by van Woerden and colleagues [11] further characterized the functional consequences of additional *TAOK1* variants in the nervous system, demonstrating a crucial role for this gene in neuronal morphology and migration. The authors described a cohort of 23 patients with 19 SNVs and 4 deleted CNVs ranging in size from 807 bp to 2 Mb. Of the nineteen SNVs, five were missense variants (p.Leu548Pro, p.Arg150Ile, p.Leu167Arg, p.Leu315Phe, p.Met231Val), seven nonsense variants (p.Gln607*, p.Lys277*, p.Glu220*, p.Arg709*, p.Arg695*, p.Arg702*, p.Arg605*), four indels (two of unknown effects on the protein, p.Lys429Asnfs*42,p.Lys78Valfs*20), and three splice site variants. The phenotypic characteristics of their cohort overlap with those described by Dulovic-Mahlow and colleagues [11] with shared predominant features including global developmental delay and muscular hypotonia. However, the authors noted that, in some cases, developmental delay maybe very mild, whereas behavioral issues and distinctive facial dysmorphisms such as frontal bossing, downslanting palpebral fissures, long philtrum, and bulbous nasal tip are widely common among *TAOK1* carriers. In addition, other significant characteristics reported in many individuals were joint hypermobility, growth anomalies (macrocephaly, overweight, and small stature), feeding difficulties during the neonatal period, and recurrent ear and airway infections. To further define the functional role of *TAOK1* in neurodevelopment, the same authors performed functional studies in vivo and in vitro. They performed in utero electroporation in mouse embryos and found that shRNA-mediated knockdown of TAOK1 at a critical time for neurodevelopment resulted in neuronal migration deficit. Hence, the reduction of *TAOK1* levels affects brain development, implying haploinsufficiency as the likely pathogenetic mechanism for *TAOK1* associated NDDs. They also tested five missense variants identified in their cohort (Table 2) by transfecting them in mouse primary hippocampal neurons. Three of them also affected TAOK1 protein expression differently (two caused a reduction, one caused an increase, and the remaining two did not affect protein expression). Similarly, neuronal morphology and migration were affected in a different way compared to wild type *TAOK1*. The p.Leu167Arg and the p.Leu315Phe variants severely impaired migration, whereas expression of the p.Leu548Pro variant resulted in increased migration. Overall, these results suggest that *TAOK1*-related NDDs can be caused by distinct pathophysiological mechanisms: haploinsufficiency for PTVs and loss of function or dominant negative mechanisms for missense variants. A similar conclusion was reached for the *SATB1* gene by Den Hoed and colleagues [7].

## 4. Concluding Remarks and Future Perspectives

Since the release of Satterstrom’s paper in February 2020 [1], many efforts have been made in order to better characterize genotype–phenotype correlations in relation to new emerging causal genes defined by the same authors as “novel genes”. Indeed, two of the novel genes we discussed above are now associated with an OMIM phenotype: *SATB1* (#619228) is associated with “developmental delay with dysmorphic facies and dental anomalies”, and *GRIA2* (#618917) is associated with “neurodevelopmental disorder with language impairment and behavioral abnormalities”. We expect that, in the coming years, new literature data will be added to the existing one. Furthermore, we would like to highlight that some of these studies (for instance [7,12]) have also concentrated on the functional characterization of some of the variant alleles found in their cohort. Preliminary results from these functional studies are very encouraging as they are shedding light on possible pathophysiological mechanisms underlying gene specific monogenic NDDs. Indeed, elucidation of pathophysiological mechanisms is of crucial importance to design targeted and personalized drug therapies. 

In addition, these studies also show the importance of performing a fully clinical assessment with particular attention to the neurological and the psychiatric characterization of the patient. Differentially specialized clinicians (neurologist, neuropsychiatrist, pediatrician, and geneticist) must collaborate together to characterize the patient phenotype in-depth.

**Figure 1:**
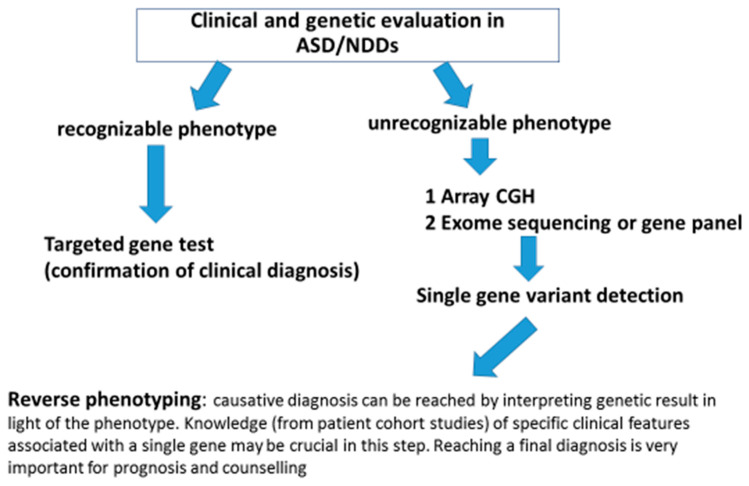
Diagnostic algorithm for genetic work up of ASD/NDD patients from clinical to molecular analysis and back.

Some specific phenotypic features are associated with at least five of the six novel genes discussed in this paper (Table 1): epilepsy is nearly always present in individuals who carry *RORB* variants; eye anomalies (globe and structural defects) are commonly found in patients with *PRR12* variants; dental anomalies and visual abnormalities are reported for *SATB1*-affected carriers; a unique behavioral phenotype characterized by sensory auditory hypersensitivity and impulsivity is typical of *RFX*-affected patients; and a neurological phenotype characterized by Rett-Syndrome-like traits, gait abnormalities, and abnormal sleep is found in *GRIA2* carriers. Unfortunately, these gene-specific phenotypic features cannot be used by the clinical geneticist to address a specific genetic test, as they are not unique to a single NDD gene but are often shared by different NDDs. Nevertheless, the reverse phenotyping data from patient cohorts and the functional data on disrupted alleles can be of great benefit for the clinicians, not just for an appropriate prescription of a genetic test but also for interpretation of the genetic results in terms of severity, prognosis, and natural history of the condition. In fact, in the current era of high-throughput technologies, it is easier (and even cheaper) to test multiple genes simultaneously rather than going gene-by-gene, regardless of the phenotype: therefore, the true challenge is not to identify the causative gene but to understand how it could be related to the clinical issues of our patients. Additionally, it is becoming frequent to detect rare variants in young children with NDD using genome or exome analysis: when the identified gene is new and there is no information about severity and prognosis, the clinical impact of the genetic results is quite low and disappoints the physician as well as the parents, because it is impossible to make any prediction about patient outcome. In this regard, clinical cohort studies focusing on a single novel gene as those reported in the present work represent a precious resource for the clinical geneticist during phenotype reevaluation as they can be of fundamental importance to reach a clinical diagnosis and make the final genotype–phenotype association. In summary, we can conclude that, with the exception of a relatively small number of conditions (such as for example Fragile X or Prader Willi syndrome), in the majority of cases, the causative diagnosis of ASD/ID cannot be reached simply by “gestalt” recognition but rather by the knowledge of specific clinical issues (see Table 1 and Table 2) reported in patients cohorts carrying single gene variants (“reverse phenotyping”). Once a pathogenetic/likely pathogenetic variant or even a VUS (variant of unknown significance) in a novel gene has been identified in a patient, checking the results of cohort studies can make the difference in genotype–phenotype correlation and parent information. Finally, in Figure 1, we propose a diagnostic algorithm for genetic work-up of ASD/NDD patients from clinical to molecular analysis and back.

## Figures and Tables

**Table 1:** The 30 novel genes and their associated functional category according to Satterstrom and colleagues [1].

Novel Gene	Category	Specific and Commonly Reported Clinical Features
SRPR	Other	_
**RORB**	Gene expression regulation	Epilepsy
DPYSL2	Cytoskeleton	-
AP2S1	Neuronal communication	-
MKX	Gene expression regulation	-
MAP1A	Cytoskeleton	-
CELF4	Gene expression regulation	-
PHF12	Gene expression regulation	-
TM9SF4	Other	-
**PRR12**	Neuronal communication	Variable structural eye defects
LDB1	Gene expression regulation	-
EIF3G	Gene expression regulation	-
KIAA0232	Other	-
VEZF1	Gene expression regulation	-
ZMYND8	Gene expression regulation	-
**SATB1**	Gene expression regulation	Severe ID and epilepsy (patients with missense variants), eye and dental anomalies
**RFX3**	Gene expression regulation	Distinct behavioral issues
PPP5C	Other	
TRIM23	Other	
ELAVL3	Gene expression regulation	
**GRIA2**	Neuronal communication	Rett-like features/hyperventilation
LRRC4C	Neuronal communication	
NUP155	Other	
PPP1R9B	Neuronal communication	
HDLBP	Gene expression regulation	
**TAOK1**	Cytoskeleton	Infant difficulties in feeding; muscular hypotonia
UBR1	Other	
TEK	Other	
CORO1A	Cytoskeleton	
HECTD4	Other	
NCOA1	Gene expression regulation	

Genes highlight in bold are those for which new information/data were found. Specific clinical features associated with these genes are also shown when present (see the text for additional information).

**Table 2:** New published studies on genotype–phenotype correlations for some of the novel genes [1].

Gene	Functional Role	New Evidence (Ref)	Number of Patients	Gene Variant	Origin	Associated Phenotype
RORB	Gene expression regulation	[2]	One patient	SNV, not specified	de novo	Eyelid myoclonia with absences, intellectual disability and ADHD
		[3]	Fourteen individuals (12 affected) Belonging to four families	c.111C>G p.Ser37Arg c.777G>T p.Trp259Cys c.96_237del141p.Gly32_Ala79del48 c.1162A>T p.Ile388Phe	inherited	Generalized and occipital epilepsy with or without ID/learning difficulties; ID only also reported
		[4]	One individual	CNV, dupl chr9:123.881.988-123.980.981	de novo	Severe ASD (non verbal, ID, and stereotypes)
PRR12	Neuronal communication	[5]	Twenty-four individuals	Twelve frameshift, six nonsense, one splice, two missense, and one gross deletion (see the manuscript for full description of variants)	de novo	Neurodevelopmental impairment (100%), variable structural eye defects (50% patients), hypotonia (61%), heart defects (52%), growth failure (54%), kidney abnormalities (35%)
		[6]	Five individuals	c.5624-2A>G p.Asp1875Glyfs*54 c.4502_4505delTGCC p.Asp1501Argfs*146 c.2353_2360delGCCGGGGG p.Ala785Profs*2 c.2045delG p. p.Gly682Aspfs*44 c.1918G>T p.Glu640* c.677dupC p.Tyr227Leufs*41 c.903_909dup p.Pro304Thrfs*46	de novo and inherited	Unilateral or bilateral microphtalmia (100%), delay (60%), short stature (40%), dysmorphic facies (40%)
SATB1	Gene expression regulation	[7]	Forty-two individuals	Thirty missense variants and ten protein-truncating variants (two nonsense, seven frameshift, and one splice)	de novo and inherited	Two clinically different NDDs: missense variants were associated with a more severe phenotype than PTVs. Subjects carrying missense variants had severe ID (57%); spasticity, hypotonia, and epilepsy were more common in subjects with missense variants. Dysmorphic features were also different between the two NDD groups. Commonly reported anomalies: eye and dental abnormalities, dysmorphic facial features
RFX gene family	Gene expression regulation	[8]	Fifteen individuals with RFX3 variants	Two frameshift variants, two splice donor variants, eight missense variants, one in-frame deletion, one 42 Kb deletion involving the last two exons of *RFX3*, and one 227 Kb deletion involving only *RFX3*	de novo (14) and inherited (1)	100% developmental delay: 72% ASD and ID of varying severity or global developmental delay in young children (78%) and ADHD (56%). Distinct behavioral features in most individuals (87%): easy excitability/overstimulation, hypersensitivity to sensory stimuli (particulary auditory), anxiety, emotional dysregulation, and/or aggression. Sleep difficulties (44%). Seizures (17%).
			Four individuals with RFX4 variants	One in-frame deletion, two missense variants, one missense variant (recessive, homozygous)	de novo (3) and inherited (1 recessive)	100% had ID or global developmental delay; 83% ASD. Seizures (33%)
			Fourteen individuals with RFX7 variants	Four frameshift variants, give stop-gain variants, one in-frame deletions, two missense variants	de novo	100% language delay; most (93%) had ID/global developmental delay; ASD (36%); ADHD (29%); in most individuals, behavioral features similar to those observed for *RFX3* individuals
GRIA2	Neuronal communication	[9]	One girl	c.1522G>T (p.Glu508Ter)	de novo	Childhood onset schizophrenia, obsessive–compulsive disorder, anxiety, and aggressive behavior
		[10]	Twenty-eight patients	-SNVs: 18 missense, 2 splice site, 1 in-frame deletion, 1 stop-gain, and 2 frameshift -three 4q32.1 microdeletions	de novo	ID, ASD, developmental delay, language impairment and striking neurological features including epilepsy or developmental epileptic encephalopathy and Rett-syndrome-like anomalies (hand washing stereotypes, regression) gait abnormalities including ataxia and dyspraxia, abnormal sleep rhythm, and irregular breathing patterns with hyperventilation episodes
TOAK1	cyotoskeleton	[11]	Eight patients	Four missense (p.Glu17Gly, p.Lys298Glu, p.Asp305Ala, p.Ser111Phe), three nonsense (p.Glu781*, p.Gln544*, p.Glu830*) and one frameshift variants (p.Leu790Phefs*3)	de novo	Broad neurodevelopmental spectrum: developmental delay (100% individuals with speech/language or motor delay), ID (50% individuals), muscular hypotonia (75% individuals), facial dysmorphisms (63%)
		[12]	Twenty-three patients	-SNVs: five missense (p.Leu548Pro, p.Arg150Ile, p.Leu167Arg, p.Leu315Phe, p.Met231Val), seven nonsense (p.Gln607*, p.Lys277*, p.Glu220*, p.Arg709*, p.Arg695*, p.Arg702*, p.Arg605*), four indels (two of unknown effects on the protein, p.Lys429Asnfs*42, p.Lys78Valfs*20) and three splice site -CNVs: four microdeletions (size range: 807 bp–2 Mb)	de novo (17), inherited (3), unknown (3)	ID/DD and/or variable learning or behavioral problems, muscular hypotonia, infant feeding difficulties, and growth problems. Common facial features include frontal bossing, downslanting palpebral fissures, long philtrum, and bulbous nasal tip

Number in brackets of column number six refers to the number of variants found for each type (e.g. de novo, inherited).

## Online Resources

The Genome Aggregation Database (gnomAD): https://gnomad.broadinstitute.org (accessed on 1 July 2021); Online Mendelian Inheritance in Man (OMIM): https://www.omim.org (accessed on 1 July 2021).
